# *Scenedesmus dimorphus* biofilm: Photoefficiency and biomass production under intermittent lighting

**DOI:** 10.1038/srep32305

**Published:** 2016-08-26

**Authors:** Andrea Efrem Toninelli, Junfeng Wang, Mingshen Liu, Hong Wu, Tianzhong Liu

**Affiliations:** 1 CAS Key Laboratory of Biofuels, Qingdao Institute of Bioenergy and Bioprocess Technology, Chinese Academy of Sciences, Qingdao, Shandong, 266101, P. R. China; 2 University of Chinese Academy of Sciences, Beijing, 100049, P. R. China; 3 State Key Laboratory of Coal-based Low Carbon Energy, Bioenergy R & D Center of ENN Sci & Tech Co., Ltd, Langfang City, Hebei, 065001, China

## Abstract

This study investigated the effect of intermittent lighting on the growth performances of a *Scenedesmus dimorphus* biofilm. Flashing light effect (FLE) is common in traditional suspended cultures of microalgae; yet, publications about this phenomenon, in the context of biofilm cultivation, are scarce. In this work we demonstrate that, thanks to intermittent illumination, it is possible for attached cultivations to fulfill FLE, improve photoefficiency and productivity as well as providing protection from photoinhibition using much lower flashing light frequencies than those usually required with suspended cultures. Medium frequency intermittent lighting (0.1 Hz) guaranteed excellent light integration resulting in 9.13 g m^−2^ d^−1^ biomass productivity, which was 8.9% higher than with continuous lighting. Results showed that a light fraction value of 0.5 always improved photoefficiency values as related to continuous light with a 118.8% maximum increase.

Microalgae are incredibly versatile unicellular organisms with a high potential for the production of biofuel and bioproducts. The biomass from microalgae can also be employed as a livestock feed, a biofertilizer, or as a soil amendment. The majority of information on algae cultivation available in the literature is related to suspended cultures, however algae cells can also be successfully grown as immobilized biofilm cultivation using a variety of reactor designs^1^,^2^,^3^,^4^,^5^,^6^. The biofilm cultivation method has some advantages on traditional suspended cultures such as higher aerial biomass productivity, reduced water use, simpler and cheaper harvest operations^7^,^8^,^9^. Regardless of the reactor design and the cultivation method employed, illumination remains the most important factor influencing the growth of microalgae^10^.

Continuous illumination is not strictly necessary to sustain cells growth. Consecutive cycles of light and darkness (intermittent illumination) can also be successfully employed to sustain the photosynthesis. In the context of suspended cultures, intermittent illumination, which happens especially at high biomass concentrations, is usually provided by the turbulent flow of the algae cells inside the reactors. Under these conditions the algae cells rapidly move from the surface of the reactor, where light intensity is very high, to the deeper portion of it, where light might be lower than the light compensation point (LCP). When the available light intensity is greater than the light saturation point (LSP), under intermittent lighting, with specific light fraction values (ε) and flash frequencies, microalgae cells may perform as if they were receiving an equivalent continuous illumination of lower intensity (CL_eq_). This phenomenon is known as “Flashing Light Effect” (FLE^11^). Many researches have demonstrated that when FLE occurred, the PE of algal cells grown under intermittent light may be higher than that of those grown with continuous light at PFD equal to that employed during the flash interval (T_F_). The term “Light Integration” (LI) is used to describe the situation where fluctuating or intermittent light is perceived as continuous, with intensity equal to the time-averaged PFD for the duration of one Light/Dark cycle time (T_C_). Therefore, when the growth performances of algae cells under intermittent light are the same as that obtainable under CL_eq_, LI has been obtained^11^,^12^. Because of this intermittent lighting and the related FLE can be considered useful tools for the protection of algae cells from physiological damage when subjected to high PFDs.

The effect of intermittent lighting on the growth performances of suspended microalgae cells has already been investigated by numerous authors, however, publications on the same topic in the context of microalgal biofilms are scarce. Due to the profound differences between the designs of the reactors employed for suspended cultures of microalgal cells and microalgal biofilms, the settings found to improve growth performances of suspensions of cells might not be appropriate with biofilm-based bioreactors. Moreover, because of the broad range of published optimal settings and sometimes contradictory results, it is difficult to extrapolate from the literature some general guidelines on how to employ intermittent lighting to take advantage of the FLE.

While in suspended cultures it is almost impossible for the algae cells to experience the same exact light exposure over time, that’s not the case when they are cultivated in the form of a biofilm. When fixed on the surface of a substrate, all of the algae cells are effectively illuminated (PFD > LCP) for up to ten days regardless of where they are located along the biofilm section^13^.

The cultivation of microalgae in the form of biofilms has attracted considerable interest in the present decade. The aim of this experiment was to investigate the influence of intermittent lighting on the growth performances of a *Scenedesmus dimorphus* attached cultivation. Various settings that define intermittent lighting were analyzed.

## Results

### Determination of saturating light intensity

A biofilm of *Scenedesmus dimorphus* algae cells was subjected to four different light intensities ranging from 80 to 400 μmol m^−2^ s^−1^. The three higher PFD values (133, 200 and 400 μmol m^−2^ s^−1^) resulted in not significantly different daily biomass productivity values (P = 0.05), higher than those induced by a PFD of 80 μmol m^−2^ s^−1^ (see Supplementary Fig. S1). Adopting a working light intensity of 400 μmol m^−2^ s^−1^ for both the CL and the intermittent light experiments, guaranteed us to be working well above LSP, determined to be at 150 μmol m^−2^ s^−1^ for *Scenedesmus dimorphus*^6^,^14^.

### Preliminary assessment of the influence of broad range variation in flashing light frequency

**Figure 1:**
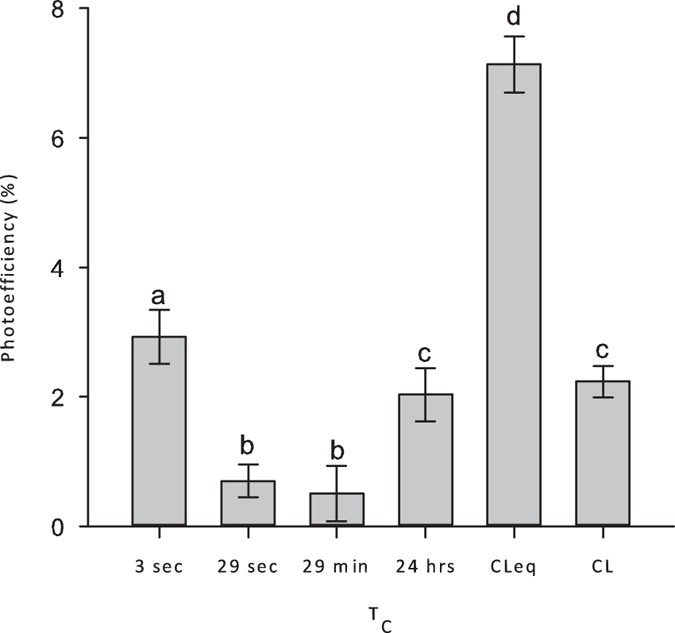
Average photoefficiency of *Scenedesmus dimorphus* subjected to different light regimens over a period of 72 hours. The light fraction value was ε = 0.2 for all of the intermittent light setups. Made exception for the one under continuous light, every other setup received the same total amount of photons; made exception for “CL_eq_”, in every other setup the PFD was set to 400 μmol m^−2^ s^−1^. Error bars represent standard deviations. Different letters represent significantly different means at P = 0.05. CL_eq_, Equivalent continuous light delivering the same total amount of light as the various intermittent light setups (PFD = 80 μmol m^−2^ s^−1^); CL, Continuous Light at 400 μmol m^−2^ s^−1^; T_C_, cycle time.

In Fig. 1 it is shown that intermittent lighting enhanced the PE of *Scenedesmus dimorphus* only at the highest tested frequency. Even though a significant improvement can be noticed over both continuous light and other intermittent light treatments (P = 0.05), the PE values are much lower than those obtained in the CL_eq_ setup. This means that LI could not be obtained and at this point both flashing light frequency and light fraction values can be further refined.

### The influence of different light fraction (ε) values

The light fraction, alone is not enough to characterize an intermittent light setup because the same value can be obtained with countless T_C_ durations. Since in the previous set of experiments (see *Results and discussion* section; subsection *Preliminary assessment of the influence of broad range variation in flashing light frequency*), the frequency range delivering best PE value was defined by T_F_ = 0.6 seconds, such value was now adopted as a measurement unit to test seven different ε values. Table 1 shows the different settings tested in this batch of experiments.

**Figure 2:**
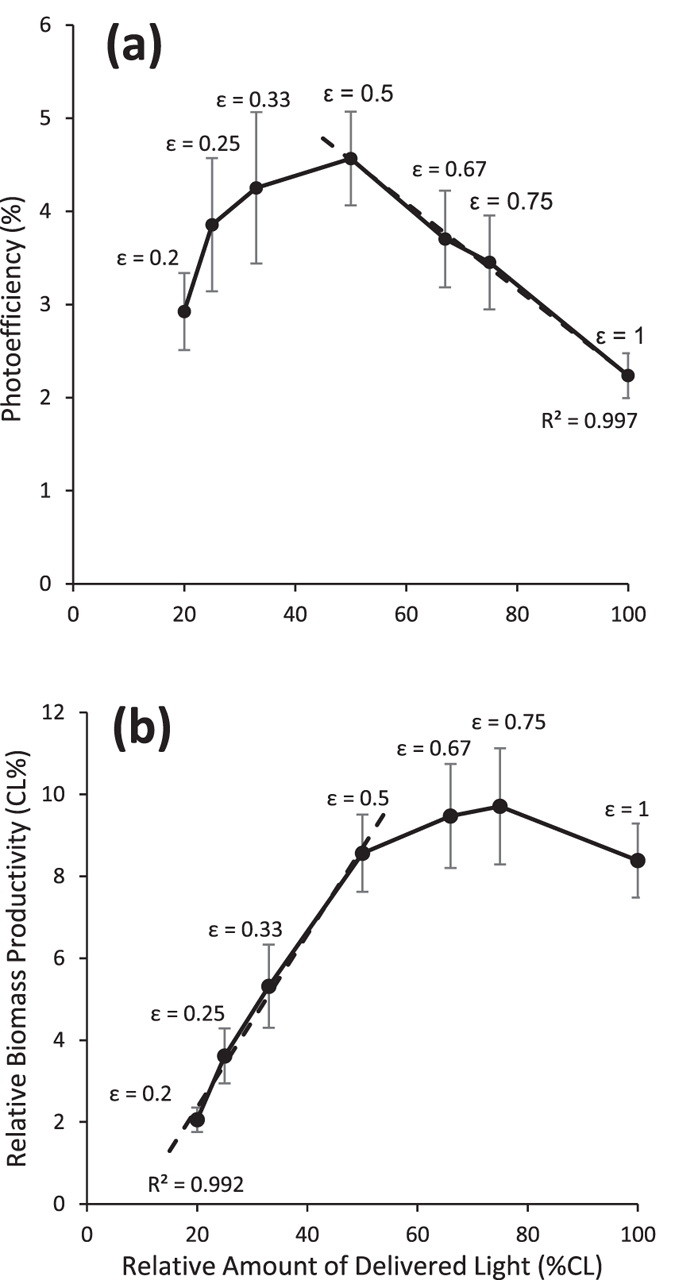
Effects of different light fraction values on average photoefficiency (**a**) and on Biomass productivity (**b**). The data are shown in relation to the total amount of light delivered under continuous light. The data are the means ± standard deviation of the values recorded during the first 72 hours following the beginning of the experiment. ε, light fraction.

With cycle times in the range of seconds, intermittent illumination improved PE values as related to continuous light, regardless of the ε value considered (Fig. 2a). The efficiency of intermittent lighting on improving the PE of *Scenedesmus d.* was at first directly proportional to the increase in the duration of T_D._ (R^2^ = 0.997). Once T_D_ started to last longer than T_F_ (ε < 0.5) though, PE values dropped dramatically, but they were still higher than those recorded under continuous light conditions.

Interestingly, given the same T_C_, the use of a longer T_D_ resulted in slightly higher PE values. The improvement in PE due to intermittent lighting was not proportional to the reduction in the total amount of delivered light; for this reason lower ε values resulted in decreased biomass productivity in comparison to the continuous light setup (Fig. 2b). While PE values were always enhanced by the use of intermittent lighting as related to the CL setup, at the tested frequencies biomass productivity was only improved with ε ≥ 0.5 (Fig. 2b). Even so, as the light fraction value decreased below ε = 0.75, the biomass productivity started to decline; slowly at first, but quite rapidly at ε < 0.5. Focusing on the influence of intermittent illumination on the PE of an attached cultivation of *Scenedesmus dimorphus*, we selected ε = 0.5 and ε = 0.33 to be further investigated in following experiments.

### The influence of flashing light frequency (refined)

Different T_C_ durations at light fraction values of 0.5 and 0.33 induced different responses in terms of both PE and biomass productivity.

**Figure 3:**
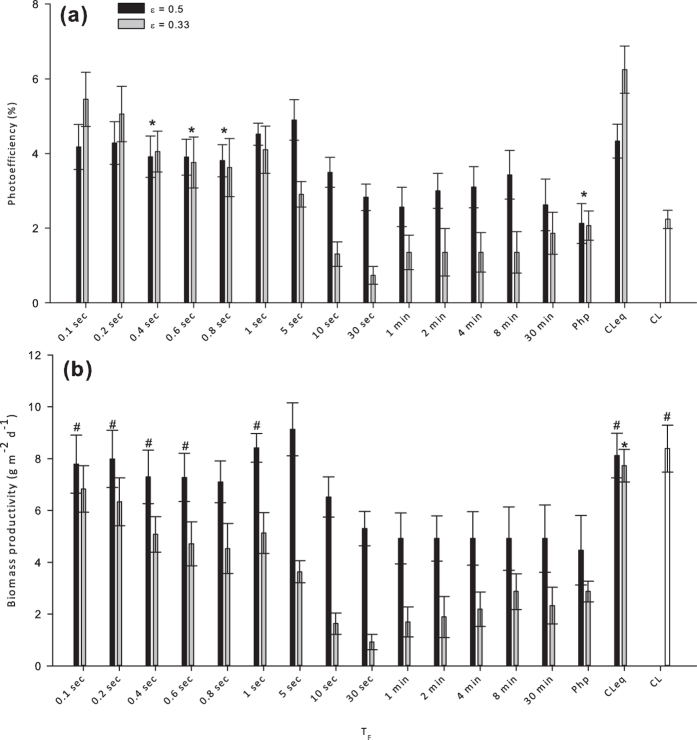
Influence of different flash light duration on *Scenedesmus dimorphus* growth performances for the light fraction values ε = 0.5 and ε = 0.33. (**a**) average photoefficiency, (**b**) average biomass productivity. Data collected during 72 hours from the beginning of the experiment have been averaged, error bars show standard deviations. Php = traditional night/day photoperiod (12 hours of darkness, 12 hours of light for ε = 0.5 and 18 hours of darkness, 8 hours of light for ε = 0.33 respectively. In both cases PFD = 400 μmol m^−2^ s^−1^); CL, Continuous Light at the same PFD used during T_F_ (400 μmol m^−2^ s^−1^); CL_eq_, Equivalent continuous light delivering the same total amount of light as the various intermittent light setups (PFD = 200 and 133 μmol m^−2^ s^−1^ for ε = 0.5 and ε = 0.33 respectively); *no significant difference at P = 0.05 between the values recorded in the two tested ε values; ^#^no significant difference at P = 0.05 among the values recorded using ε = 0.5.

In terms of PE the algae cells responded quite differently to the two light fractions under investigation. Higher values were obtained at higher frequencies with ε = 0.33; however, at lower frequencies ε = 0.5 offered better performances (Fig. 3a). Moreover, only with ε = 0.5 intermittent lighting always increased PE values as compared to CL; for T_F_ values between 10 seconds and 30 minutes, the use of ε = 0.33 caused the PE of *Scenedesmus dimorphus* to be up to 67% lower than that recorded under CL. The maximum PE value reached 5.45% with ε = 0.33 and T_F_ = 0.1 seconds; this was more than 10% higher than the maximum PE value obtained using ε = 0.5 (T_F_ = 5 seconds). Both ε = 0.33 and ε = 0.5 produced not significantly different PE values (P = 0.05) using T_F_ durations between 0.4 and 0.8 seconds as well as with the traditional day/night setup.

Globally, faster flashing light frequencies resulted in higher light use efficiency. Considering the results shown in the *Results and discussion* section; subsection *Preliminary assessment of the influence of broad range variation in flashing light frequency*, this kind of response to the high frequency was expected. Anyway, even though ε = 0.33 allowed for higher PE values at the highest tested frequency, LI was obtained only with ε = 0.5 using T_F_ durations between 0.1 and 0.6 seconds and between 1 and 5 seconds. Surprisingly, when T_F_ lasted 0.8 seconds the biomass productivity of *S. dimorphus* was significantly lower (P = 0.05) and LI could not be obtained.

Biomass productivity values are generally higher using ε = 0.5 (Fig. 3b). The difference between the data recorded with the two tested light fraction values is lower at the extremes of the tested frequency range and higher when TF lasts between 5 and 30 seconds. Light integration could generally be obtained with T_F_ values between 0.1 and 5 seconds, but only if ε = 0.5. The maximum biomass productivity reached 9.13 g m^−2^ d^−1^ using ε = 0.5 and T_F_ = 5 seconds; this is 11% higher than under CL_eq_ and more than 25% higher than the maximum biomass productivity obtained with ε = 0.33. Confirming the data from the previous set of experiments (see *Results and discussion* section; subsection *The influence of different light fraction* (*ε*) *values* and Fig. 2), inside the medium-high frequency range (T_F_ between 0.4 and 0.8 seconds), both ε = 0.33 and ε = 0.5 did not induce significantly different results in terms of PE (P = 0.05); as the flashing light frequency further increased, using a lower light fraction value induced higher light use efficiency reaching a maximum of 5.45% (based on visible light; T_F_ = 0.1 seconds).

Even though the use of intermittent illumination can improve the light use efficiency of *S. dimorphus*, as related to the CL setup, high PE values do not always translate into high net biomass yields. The increase of light use efficiency detected was not proportional to the reduction in total amount of delivered photons and ultimately resulted in lower biomass productivity values (Fig. 3b). Higher net biomass yields were obtained using T_F_ and T_D_ of equal duration.

## Discussion

Numerous researchers already experimented with flashing light; however, it is not always easy to compare the results from different studies because these seem to change depending on the algae species considered and other set variables. Terry^12^ obtained LI with frequencies close to 1 Hz using *Phaeodactylum tricornutum*. Sforza *et al*.^15^ working with *Nannochloropsis sp.* reported that 1 Hz and 5 Hz flashing light frequencies with R_L/D_ = 1/9 did not allow to obtain LI, but at higher flashing light frequencies that would be possible. Nedbal *et al*.^16^ found that, for *Chlorella vulgaris,* LI in terms of oxygen evolution rate was possible with T_F_ shorter than 1 millisecond using R_L/D_ of 1/5 and 1 (CL_eq_ = 500 μmol m^−2^ s^−1^). Slower frequencies (T_F_ between 50 microseconds and 200 milliseconds) still induced LI, but this time that happened in terms of growth rate, not oxygen evolution rate. In our case LI could only be achieved at much lower frequencies than those previously reported, and only when T_D_ did not last longer than T_F_.

Bioreactors based on attached cultivation are drastically different from traditional ones where algae cells are cultivated suspended into a liquid environment. Since microalgae cells in biofilm bioreactors are fixed on the surface of a substrate, it is much harder to make use of intermittent lighting using sunlight as the sole illumination source without them suffering from photo-inhibition. One way to obtain intermittent lighting on a biofilm bioreactor could consist in cultivating the algae cells on top of a substrate that would constantly move from a dimly lit area to a brighter one. Revolving belts^1^, rotating spools^2^ and rotating disks^17^ are possible examples of how to achieve that. The ability to use lower flashing light frequencies, while still being able to improve PE values and protect algae cells from photoinhibition and physiologic damage, could translate into a lower energy input required to set the reactors’ modules in motion and obtain intermittent lighting. An increased ratio between the cultivated surface area and the reactor’s footprint might further increase the productivity of the system.

Intermittent lighting is defined by: (1) the duration of a complete Light/Dark cycle (T_C_) intended as the sum of the duration of the photoperiod (T_F_) and that of the scotoperiod (T_D_), and (2) by the ratio between T_F_ and T_D_ (R_L/D_). This last factor can also be expressed in terms of *light fraction* (ε) intended as the ratio between T_F_ and T_C_. The duration of T_C_ determines the frequency of the intermittent lighting, viz. the lower the T_C_ value, the higher the flashing light frequency.

Theoretically, when aiming to modulate intermittent illumination, one might be tempted to merely consider the speed at which photosynthetic reactions occur. In this way the ideal frequency and ratio between the flash interval and the dark interval should keep pace with both the frequency and duration of the light-reactions and the light-independent reactions of photosynthesis, which usually occur in the range of milliseconds and seconds to hours respectively^15^,^18^. However, while some studies support this theory^19^,^20^, some others show that photoperiods as long as 0.1 seconds^21^, 2.02 ± 0.19 seconds^22^ or 5.8 minutes^23^ followed by dark intervals could still induce an increase in cell density, as compared to continuous light. The ability to use frequency values on a lower order of magnitude than those theoretically required to run the photosynthetic processes, while still inducing FLE, would be of great help to the mass cultivation of microalgae biofilms since lower energy input would be necessary to obtain them. Our choice of using a PFD of 400 μmol m^−2^ s^−1^ as a saturating light intensity is supported by Liu *et al*.^6^, Gris *et al*.^14^ and Chen *et al*.^23^. Liu *et al*.^6^ and Gris *et al*.^14^ reported that, regardless of the cultivation technique adopted, the growth rate of *Scenedesmus sp.* could not increase any further above PFD values of 150 μmol m^−2^ s^−1^ meaning that LSP had been reached. Chen *et al*.^23^ almost doubled the biomass productivity of the same microalgal species using intermittent illumination at 400 μmol m^−2^ s^−1^.

Interestingly though, when we used the same algal species, ε value, PFD, and flashing light frequency employed by Chen *et al*.^23^, intermittent lighting not only failed to increase biomass productivity as compared to the continuous light setup, but it also had a strong negative effect. Higher flashing frequencies, at the same light fraction value, performed better than both the CL and the Php setups, however, LI could not be reached. This may be due to the fact that microorganisms tend to behave differently when cultivated as a biofilm, instead of a suspended culture of cells. First of all, as mentioned previously, suspended cells experience light exposure differently from those in a biofilm. Secondly, the microenvironment surrounding the cells can be totally different; variations in nutrient concentration, distances among cells and possible interaction between them may induce different growth performances between freely dispersed cells and those growing attached to a substrate.

Another explanation to our inability to recreate the results of Chen *et al*.^23^ might have been offered by Crill^24^ and Pulz^25^ according to whom light-acclimation occurs on a timescale longer than light-inhibition. In his mini-review on photobioreactors, Pulz^25^ reported that while as much as 10 to 40 minutes are required for the algae cells to photoadapt to high PFDs, irreversible destruction can happen in a matter of a few minutes, with more than 50% damage after only 10 to 20 minutes. In photoautotrophic conditions, photons of light are the main source of energy for the growth of algae cells. When irradiance is too high the photosynthetic capacity is reduced because of photoinhibition and, if the same condition persist over time, it is usually followed by photodestruction of photosynthetic pigments^26^,^27^,^28^. Phytoplankton’s response to the exposure to high PFD (photoacclimation) is much slower than the rate at which light is absorbed and assimilate by the cell^29^. If algae cells are subjected to an inhibiting PFD, the net photosynthetic rate will quickly start to decline^30^. If light intensity is too low instead, it has been demonstrated, both with a simulated virtual photobioreactor^31^ and with cultures used for physiological research^32^ that microalgae cells’ logarithmic growth will not prevail. When the PFD is insufficiently high to support cells’ growth and no metabolizable organic carbon can be found in the medium, algae cells will metabolize their own cell components to obtain maintenance energy, thus leading to a decrease in cell weight^33^. In view of this it is of the utmost importance to identify the optimal ε value and flashing light frequency that allow microalgae cells to use energy light in the most efficient way.

In our case the algal cells experiencing T_C_ of 29 seconds and 29 minutes (T_F_ = 5.8 seconds and 5.8 minutes respectively) might not have been able to photo-adapt to the high light intensity they were receiving before the LED were switched off and in the meanwhile, they already suffered photoinhibition induced damage that ultimately resulted in low PE values. When T_C_ lasted 24 hours instead, the algae cells were exposed to light for a few hours; such long photoperiod may have allowed them to adapt to the high light intensity, therefore they performed more efficiently than with T_F_ in the range of seconds or minutes even though the same total amount of photons was delivered over 24 hours. This confirmed previous studies on the FLE reporting higher PE values at higher flashing light frequencies^12^,^20^,^23^,^34^,^35^ and also required us to investigate a range of different T_C_ durations as well as light fraction values. Light fraction values of 0.5 and 0.33, resulted in photoefficiency of the algae cells respectively 118.8 and 143.3% higher than under continuous light. A flash duration of 5 seconds, with light fraction equal to 0.5, delivered a biomass productivity of 9.13 g m^−2^ d^−1^; 8.9% higher than under continuous light. Even though maximum photoefficiency was obtained with light fraction 0.33, light integration was only possible with light fraction of 0.5 (flash duration between 0.1 and 5 seconds). Such a limited improvement in biomass productivity in comparison with continuous lighting is not extremely encouraging for scale-up and industrial applications. However, we demonstrated on an attached cultivation of *Scenedesmus dimorphus* that it is possible to fulfill flashing light effect using intermittent lighting frequencies much lower than those required in suspended cultures. In addition to intermittent exposure to solar radiation, when outdoors, light dilution^6^ could also be employed to further improve the biomass productivity of a biofilm-based bioreactor. Liu *et al*.^6^ obtained aerial productivities up to 7 times higher than those of a conventional open pond when they increased the ratio between the cultivated surface and the land surface occupied by the reactor by a factor of 10. Additional research is still needed to verify whether is it possible to simultaneously take advantage of both the flashing light effect and the light dilution concept as a way to obtain on a contained surface high amounts of biomass easily harvestable. As mentioned previously, it is difficult to extrapolate from the literature some general guidelines on the use of intermittent light as a mean of improving the biomass productivity of microalgal biofilm cultivations. For this reason, future research will test the effect of intermittent light on other species cultivated in the form of a biofilm in order to verify whether the response to intermittent lighting in this context could be generalized.

## Methods

### Algal strain and inoculum preparation

The fresh water microalga *Scenedesmus dimorphus* used in this research was obtained from the Key laboratory of Biofuel (Qingdao, China) and maintained in BG11 medium. The inoculum cells for the attached cultivation, were multiplied inside glass bubbling columns (inner diameter is 5 cm containing 700 ml of BG11 medium) with aeration of 2% (v/v) CO_2_ enriched compressed air at the rate of 60 L min^−1^. The broth temperature was maintained at 22 ± 2 °C during cultivation. Light intensity at the surface of the column was 100 μmol m^−2^ s^−1^ (6000–6500 °K 28 w fluorescent lamps, NFLYZ28-T5, NVC, China). The algae cells were cultivated inside the glass columns for 7 days before being used to inoculate the biofilm reactor.

### Biofilm cultivation system

**Figure 4:**
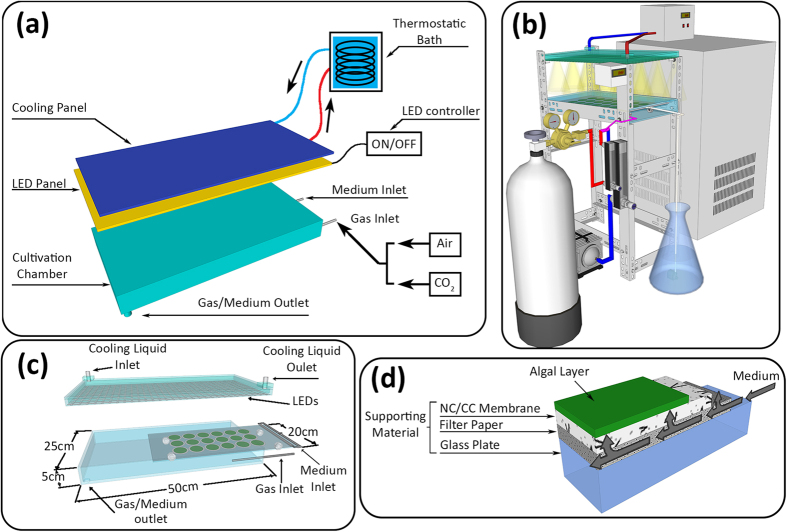
Schematic and graphical representation of the experimental set-up. During the cultivation, the entire bench was wrapped with a black polyethylene film in order to block any unwanted environmental light from reaching the algae disks. (**a**) diagrammatic representation of the experimental set-up. The cultivation chamber is illuminated by an LED board connected to a controller that defines the intermittent light settings. A cooling panel connected to a thermostatic bath is installed on the back of the LED board. CO_2_ enriched air is inflated inside the cultivation chamber. Growth medium is not recycled. (**b**) Graphical representation of the experimental set-up: general view. (**c**) Detail of the biofilm reactor cultivation chamber and cooled LED board. The glass plate holding the algae disks can slide outside the cultivation chamber allowing the collection of samples. Cling film seals the cultivation chamber before and after collection of samples. (**d**) Detailed section view. The glass plate offers a rigid support to the filter paper wetted by the medium. This flows by gravity from right to left and moves upwards by capillary action through the nitrocellulose membrane providing water and nutrients to the algae cells.

Figure 4 shows diagrammatic and graphical representations of the experimental setup. The growth chamber for the biofilm cultivation consisted in a transparent glass box measuring, internally, 50 cm × 25 cm × 5 cm (0.5 cm thickness) and missing one of the two smallest faces. The reactor laid on a wide face, tilted about 4° on the longitudinal axis to allow gravitational flow of the growth medium across the cultivated surface. One small hole was opened on the floor plate of the reactor for drainage of the used growth medium and for gas exchange. During experiments, the open side of the box was sealed using cling film to guarantee an efficient delivery of the CO_2_ enriched compress air to the algae cells. The inside of the glass growth chamber was aerated with compressed air enriched with 1% CO_2_ (v/v) at a speed of 10 L min^−1^ so to deliver a non-limiting CO_2_ supply.

A glass plate (20 cm × 45 cm) was positioned inside the glass box as a support for the attached cultivation. A polyurethane pipe (0.4 cm inner diameter) was fitted on the top brim of the support plate to act as the medium distributor. The support glass plate and the polyurethane pipe were both covered by one sheet of common filter paper. The nutrient medium was delivered with a submersible pump from an Erlenmeyer flask. During the cultivation of the biofilm, BG11 medium dripped between the support glass plate and the filter paper reaching the algae cells by capillary action. The flow rate of the culture medium was carefully regulated at 30.4 ± 4.5 ml h^−1^. The amount of BG11 delivered to the growth chamber was kept low enough to prevent any visible wash-off of the cultivated cells from the biofilm. In order to maintain the nutrients level constant during the cultivation, the effluent medium was discarded directly without any recirculation.

Fifteen LED strips (12 v, 6000-6500K; Shenzhen GreeThink Electronic Co., Ltd.) were installed on a 50 × 25 × 0.3 cm glass plate. The light unit was positioned above and parallel to the growth chamber (Fig. 4c); light intensity was regulated by adjusting the distance between the light panel and the cultivated surface. Intermittent lighting was provided by connecting the LEDs to a digital time relay (DH48S-S, Shanghai OFO Electrical Co., LTD). In order to prevent the LEDs from overheating, the glass plate holding the LEDs was connected to a cooling system (Fig. 4). The fluid circulating inside the cooling system was maintained at a temperature of 17 ± 0.5 °C by means of a recycling thermostatic bath (DL-4005, Ningbo Haishu Sklon Electronic Instrument Co., Ltd., China).

Cellulose acetate/nitrate membranes (pore size 0.45 μm) were inoculated via filtration of a determined amount of suspended cells from the bubbling column. The cells gathered onto the cellulose acetate/nitrate membrane formed an algal ‘disk’ with 12.3 ± 0.5 cm^2^ of footprint and approximately 9 g m^−2^ dry biomass inoculum density. Multiple algae ‘disks’ were put on the filter paper arranged close one to another in 6 rows and 3 columns leaving an empty space of equal distance toward the edges of the support plate (Fig. 4d).

The air temperature inside the glass box was 22 ± 2 °C. During the cultivation of cells, the entire bench holding the growth chamber and the light panel was wrapped with a black polyethylene film in order to block any unwanted environmental light from reaching the algae disks. No obvious contaminations were found during each batch of experiment (72 hours).

### Determination of a saturating photon flux density

Given the employed light source, the highest PFD we could reach without causing condensation on the inner surface of the growth chamber was 400 μmol m^−2^ s^−1^. Condensation inside the roof of the growth chamber would have created big drops of water that could both alter the light distribution on the cultivated surface and suddenly detach washing cells off of the algal disks. Four sets of “algal disks” were subjected for 72 hours to PFD levels of 80, 133, 200 and 400 μmol m^−2^ s^−1^ respectively and the biomass productivities (referred to the cultivated surface area) were then compared to determine whether the LSP had been reached. Such light intensities corresponded respectively to those employed with intermittent light at ε = 0.2; ε = 0.33; ε = 0.5 and ε = 1 with 400 μmol m^−2^ s^−1^ during the flash interval. PFDs were measured with a light meter (LI-250A, LI-COR, Inc., US.) equipped with a quantum sensor.

### Preliminary assessment of the influence of broad range variation in flashing light frequency

We refined the intermittent light settings starting from those employed by Chen *et al*.^23^. In an experiment on forced light/dark circulation of a suspended culture of *Scenedesmus dimorphus* they concluded that with ε = 0.2 and cycle time ≈ 29 minutes it was possible to almost double the biomass productivity as compared to a conventional open pond. Other authors also agree that T_D_ should last longer than T_F_ for FLE to take place^12^,^14^,^20^,^35^. As a preliminary test, the ratio between the flash interval (T_F_) and the dark interval (T_D;_ R_L/D_) was set to 1/4 with T_F_ = 5.8 minutes as in Chen *et al*.^23^. Given the same R_L/D_, other employed T_F_ values were in the range of milliseconds (T_F_ = 600 milliseconds), seconds (T_F_ = 5.8 seconds) and hours (T_F_ = 4.8 hours) respectively. Due to technical limits of the timer employed and given the chosen R_L/D_, it was not possible to test cycle times shorter than 3 seconds. Since R_L/D_ and PFD were set constant among the four tests, they all received the same photon flux and illumination time over the course of 24 hours.

### The influence of light fraction values

*S. dimorphus* cells were subjected to 7 different light fraction values in order to verify the influence of different T_D_ durations on biomass productivity and PE. The employed T_D_ were multiples of T_F_. Reciprocal R_L/D_ values were also tested to verify whether, given the same T_C,_ the cells would perform best being subjected to a longer T_D_^12^,^14^,^20^,^35^ or to a longer T_F_. PFD was set to 400 μmol m^−2^ s^−1^. Table 1 describes in better detail the settings tested in this batch of experiments.

### The influence of intermittent light frequency values

The effect of different flashing light frequencies on the growth performances of *Scenedesmus dimorphus* was investigated.

The data recorded under intermittent illumination were compared with those obtained from three other experimental setups. The first one was provided with continuous light at the same PFD as for the flash interval in the intermittent lighting setups; the second and the third one received the same total amount of photons as the flashing light experiments, but in the form of: 1) a traditional night/day lighting regime (PFD of 400 μmol m^−2^ s^−1^; Php), and 2) of time-averaged light intensity (CL_eq_) respectively. The comparison with samples receiving CL_eq_ was necessary to determine whether LI could be achieved or not. Table 2 describes in better detail the characteristics of the intermittent lighting tested in this batch of experiments.

All the experiments were performed in triplicate to reduce sampling errors and ensure repeatability.

### Biomass yield and biomass productivity of biofilm cultivation

Biomass yield and biomass productivity of biofilm cultivation were measured according to Liu *et al*.^6^. Three samples were removed from random position inside the growing chamber. The first set of samples was collected twenty-four hours after the lights had been switched-on. After that, collection of samples was carried out every twelve hours until the end of the experiment (72 hours after the lights had been first switched-on).

### Photoefficiency

Photoefficiency, based on visible light, was obtained as follows:


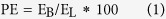


Where the energy contained in the net biomass production (E_B_) and the energy delivered with light (E_L_) were calculated based on the following: the energy contained in one gram of unstressed dried biomass, usually amounts to 20–23 kJ^36^ (we considered it to be 20 kJ g^−1^); 48% of the total solar radiation falls into the range of visible light^37^; in average, one mole of visible photons contains 217 kJ of energy^36^.

### Statistical analysis

The results are reported as average ± standard deviation. Single factor analysis of variance (ANOVA) was used to ascertain significant differences in biomass yield, biomass productivity or photosynthetic efficiency. Tukey’s range test followed ANOVA to determine significant differences among treatments. The level of statistical significance was P < 0.05. The Microsoft Excel Data Analysis ToolPak (Microsoft Corporation, 2013) was used for all statistical analysis.

## Additional Information

**How to cite this article**: Toninelli, A. E. *et al*. *Scenedesmus dimorphus* biofilm: Photoefficiency and biomass production under intermittent lighting. *Sci. Rep.* **6**, 32305; doi: 10.1038/srep32305 (2016).

## Supplementary Material

Supplementary Information

## Figures and Tables

**Table 1:** Description of intermittent light conditions employed to determine the effect of different light fraction values on the growth performances of a *Scenedesmus dimorphus* attached cultivation.

ε	T_F_ (sec)	Frequency^a^
0.20	0.6	1200
0.25	0.6	1500
0.33	0.6	2000
0.50	0.6	3000
0.67	1.2	2000
0.75	1.8	1500
1.0	CL	/

^a^Frequency values are intended as “cycles per hour”. ε, light fraction; T_F_, flash duration; CL, continuous light.

**Table 2:** Description of intermittent light conditions employed to determine the effect of the duration of the flash interval on the growth performances of a *Scenedesmus dimorphus* attached cultivation.

T_F_	Frequency^a^	
	ε = 0.5	ε = 0.33
30 min	1	0.67
8 min	3.75	2.50
4 min	7.50	5
2 min	15	10
1 min	30	20
30 sec	60	40
10 sec	180	120
5 sec	360	240
1 sec	1800	1200
0.8 sec	2250	1500
0.6 sec	3000	2000
0.4 sec	4500	3000
0.2 sec	9000	6000
0.1 sec	18000	12000

^a^Frequency values are intended as “cycles per hour”.
