# Supplementary material for: Scenedesmus dimorphus biofilm: Photoefficiency and biomass production under intermittent lighting
Source: Sci Rep. 2016 Aug 26;6:32305. doi: 10.1038/srep32305 (PMC4999893; doi:10.1038/srep32305)
Supplement: Supplementary Information [file srep32305-s1.pdf]

Scenedesmus dimorphus biofilm: Photoefficiency and biomass production under intermittent lighting

Andrea Efrem Toninelli[1,2], Junfeng Wang[2,*], Mingshen Liu[3], Hong Wu[3], Tianzhong Liu[2,*]

[1] University of Chinese Academy of Sciences, Beijing, P. R. China, 100049

[2] CAS Key Laboratory of Biofuels, Qingdao Institute of Bioenergy and Bioprocess Technology, Chinese Academy of Sciences, Qingdao, Shandong, P.R. China, 266101

[3] State Key Laboratory of Coal-based Low Carbon Energy, Bioenergy R&D Center of ENN Sci & Tech Co., Ltd, Langfang City, Hebei, China, 065001

[*] Corresponding author; Correspondence and request for materials should be addressed to T. L. (Tel/fax: +86 532 8066 2735. E-mail: liutz@qibebt.ac.cn)

Supplementary Figure S1

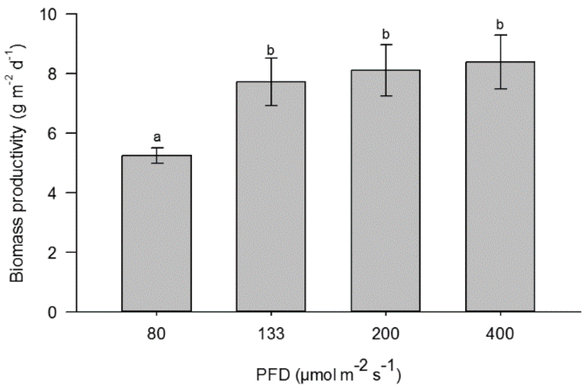

Supplementary Figure S1: Average biomass productivity at different PFD values. Data are the means of three replicates and error bars show standard deviations. Different letters indicate significantly different means at P= 0.05.
